# Sepsis-induced coagulopathy (SIC) in the management of sepsis

**DOI:** 10.1186/s13613-025-01441-3

**Published:** 2025-02-19

**Authors:** Yun Ji

**Affiliations:** Department of Surgical Intensive Care Unit, The Second Affiliated Hospital, School of Medicine, Zhejiang University, 88 Jiefang Road, Hangzhou, Zhejiang China

Dear Editor,

I read with great interest the article by Iba et al. published in the *Annals of Intensive Care* regarding sepsis-induced coagulopathy (SIC) [1]. The article provides a detailed introduction to the criteria for SIC and its scoring system, offering valuable insights for better diagnosis and possibly management of sepsis-related disseminated intravascular coagulation (DIC). The authors emphasize the clinical benefits of the SIC scoring system, which include early identification of DIC, development of treatment strategies, monitoring of disease progression, risk stratification, and enhanced prognostication.

Currently, the diagnostic criteria for SIC rely on platelet counts, prothrombin time/international normalized ratio (PT-INR), and the modified Sequential Organ Failure Assessment score. Given the availability of more specific and sensitive hemostatic assays, conventional coagulation markers, such as platelets and PT-INR, can be integrated with these assays to provide a more comprehensive assessment of coagulopathy. Viscoelastic tests, such as thromboelastography (TEG) or rotational thromboelastometry (ROTEM), which have been considered in the setting of SIC for years, offer several significant advantages over conventional coagulation tests [2]. First, these tests offer a significantly shorter turnaround time for results—some parameters can be assessed in as little as 2 min—making them particularly suitable for critical care settings. Second, they allow for real-time visualization of clot formation at the point of care through what are known as temograms. Third, viscoelastic tests analyze whole blood samples, unlike conventional coagulation tests that use only plasma, enabling a comprehensive evaluation of the interactions between clotting factors, platelets, and red blood cells. However, they also present challenges such as relatively high costs, data interpretation that is not simple, issues with standardization, and the complexity of operating the equipment, which requires specialized training [2].

Therefore, combining viscoelastic testing with conventional coagulation indicators could enhance the accuracy of coagulopathy diagnoses. Many studies have indicated that these methods can effectively diagnose sepsis-related coagulopathies, including hypercoagulable and hypocoagulable states, which are often associated with DIC and microvascular thrombosis (Table 1) [3–7]. Previous studies and a systematic review suggest that the alpha angle (TEG), maximum amplitude (TEG), and maximum clot firmness (ROTEM) may serve as specific markers for SIC [3–8]. Future research should explore the potential for establishing diagnostic criteria for SIC based on viscoelastic testing.

**Table 1:** Selected studies on viscoelastic tests and sepsis-induced coagulopathy (*n* ≥ 100)

First author, year	Study design	Population (*n*)	TEG or ROTEM	Timing of measurement	Conclusions
Bui-Thi, 2023 [3]	Prospective, observational	Sepsis and septic shock (161)	ROTEM	Within 24 h of admission	ROTEM can differentiate coagulopathy phenotypes, including hypocoagulability, hypercoagulability, hyperfibrinolysis, and mixed disorders exhibiting both hypercoagulability and hypocoagulability.
Kim, 2022 [4]	Retrospective observational	Septic shock patients with non-overt DIC (295)	TEG	At the time of septic shock recognition	An MA < 64 mm was independently associated with DIC development.
Kim 2021 [5]	Prospective observational	Septic shock patients with normal PT and aPTT (414)	TEG	At the time of septic shock recognition	Septic shock patients with normal PT and aPTT may have an impaired TEG profile, such as a hypocoagulable state, which is linked to increased mortality.
Davies 2018 [6]	Prospective observational	Sepsis, severe sepsis and septic shock (100)	ROTEM	Within 24 h of admission	ROTEM indicated enhanced clot structural development in sepsis and severe sepsis, suggesting a hypercoagulable state. In septic shock, ROTEM showed impairment of fibrinolysis despite a normocoagulable state.
Haase, 2015 [7]	Prospective cohort	Severe sepsis (260)	TEG	At time of inclusion and the following 5 days	Hypocoagulability measured by TEG is linked to higher mortality and bleeding risk.

aPTT: activated partial thromboplastin time; CI: confidence interval; DIC: disseminated intravascular coagulation; MA: maximum amplitude; OR: odds ratio; PT: prothrombin time; ROTEM: rotational thromboelastometry; TEG: thromboelastography

In conclusion, although Iba et al. [1] provided valuable insights into SIC, further research is essential, particularly in applying viscoelastic testing to SIC and exploring their potential value in diagnosing DIC in sepsis.

## Data Availability

Not applicable.

## References

[1] Iba T, Helms J, Levy JH. Sepsis-induced coagulopathy (SIC) in the management of sepsis. Ann Intensive Care. 2024;14(1):148. 10.1186/s13613-024-01380-5 PMC11415329 39302568

[2] Hartmann J, Hermelin D, Levy JH. Viscoelastic testing: an illustrated review of technology and clinical applications. Res Pract Thromb Haemost. 2023;7(1):100031. 10.1016/j.rpth.2022.100031 PMC9903681 36760779

[3] Bui-Thi HD, Le Minh K. Coagulation profiles in patients with sepsis/septic shock identify mixed hypo-hypercoagulation patterns based on rotational thromboelastometry: A prospective observational study. Thromb Res. 2023;227:51 − 9. 10.1016/j.thromres.2023.05.010 37235948

[4] Kim SM, Kim SI, Yu G, Kim YJ, Kim WY. Which Septic Shock Patients With Non-Overt DIC Progress to DIC After Admission? Point-of-Care Thromboelastography Testing. Shock (Augusta, Ga). 2022;57(2):168 − 74. 10.1097/SHK.0000000000001847 35025842

[5] Kim SM, Kim SI, Yu G, Kim JS, Hong SI, Chae B, et al. Role of thromboelastography in the evaluation of septic shock patients with normal prothrombin time and activated partial thromboplastin time. Sci Rep. 2021;11(1):11833. 10.1038/s41598-021-91221-3 PMC8178375 34088928

[6] Davies GR, Lawrence M, Pillai S, Mills GM, Aubrey R, Thomas D, et al. The effect of sepsis and septic shock on the viscoelastic properties of clot quality and mass using rotational thromboelastometry: A prospective observational study. J Crit Care. 2018;44:7–11. 10.1016/j.jcrc.2017.09.183 28988002

[7] Haase N, Ostrowski SR, Wetterslev J, Lange T, Møller MH, Tousi H, et al. Thromboelastography in patients with severe sepsis: a prospective cohort study. Intensive care medicine. 2015;41(1):77–85. 10.1007/s00134-014-3552-9 25413378

[8] Hu YL, McRae HL, Refaai MA. Efficacy of viscoelastic hemostatic assay testing in patients with sepsis-induced disseminated intravascular coagulation. Eur J Haematol. 2021;106(6):873-5. 10.1111/ejh.13617 33682140

